# Supplementary material for: Histone Tail Dynamics in Partially Disassembled Nucleosomes During Chromatin Remodeling
Source: Front Mol Biosci. 2019 Nov 28;6:133. doi: 10.3389/fmolb.2019.00133 (PMC6896900; doi:10.3389/fmolb.2019.00133)
Supplement: Supplementary file 1 [file Data_Sheet_1.pdf]

Supplementary Material

SUPPLEMENTARY FIGURES

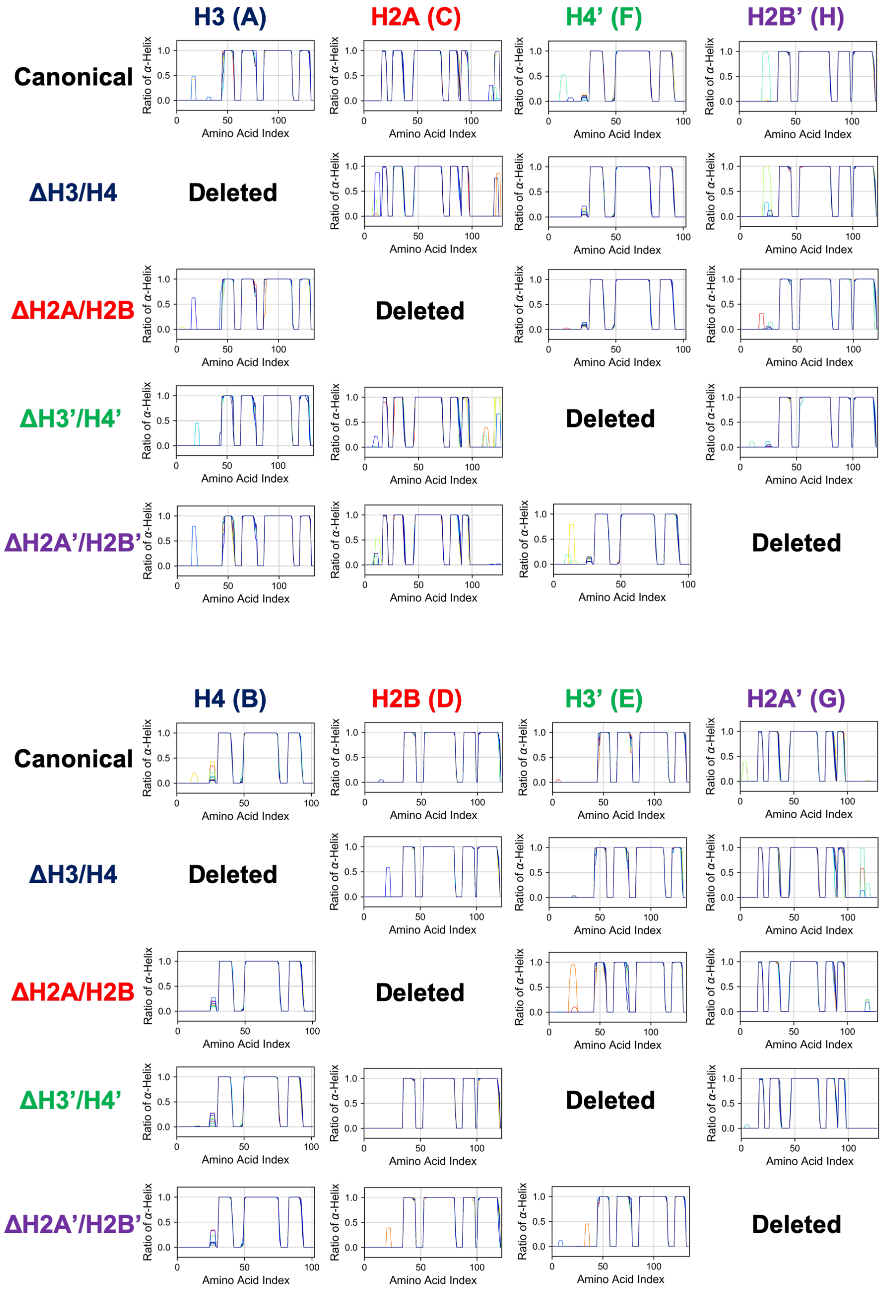

Figure S1. Secondary Structure Stability of Histones in Each Nucleosome. Horizontal and vertical axes of each graph show amino acid index and ratio of α-helix formation in each histone, respectively. Colors show different simulation trajectories (total 10).

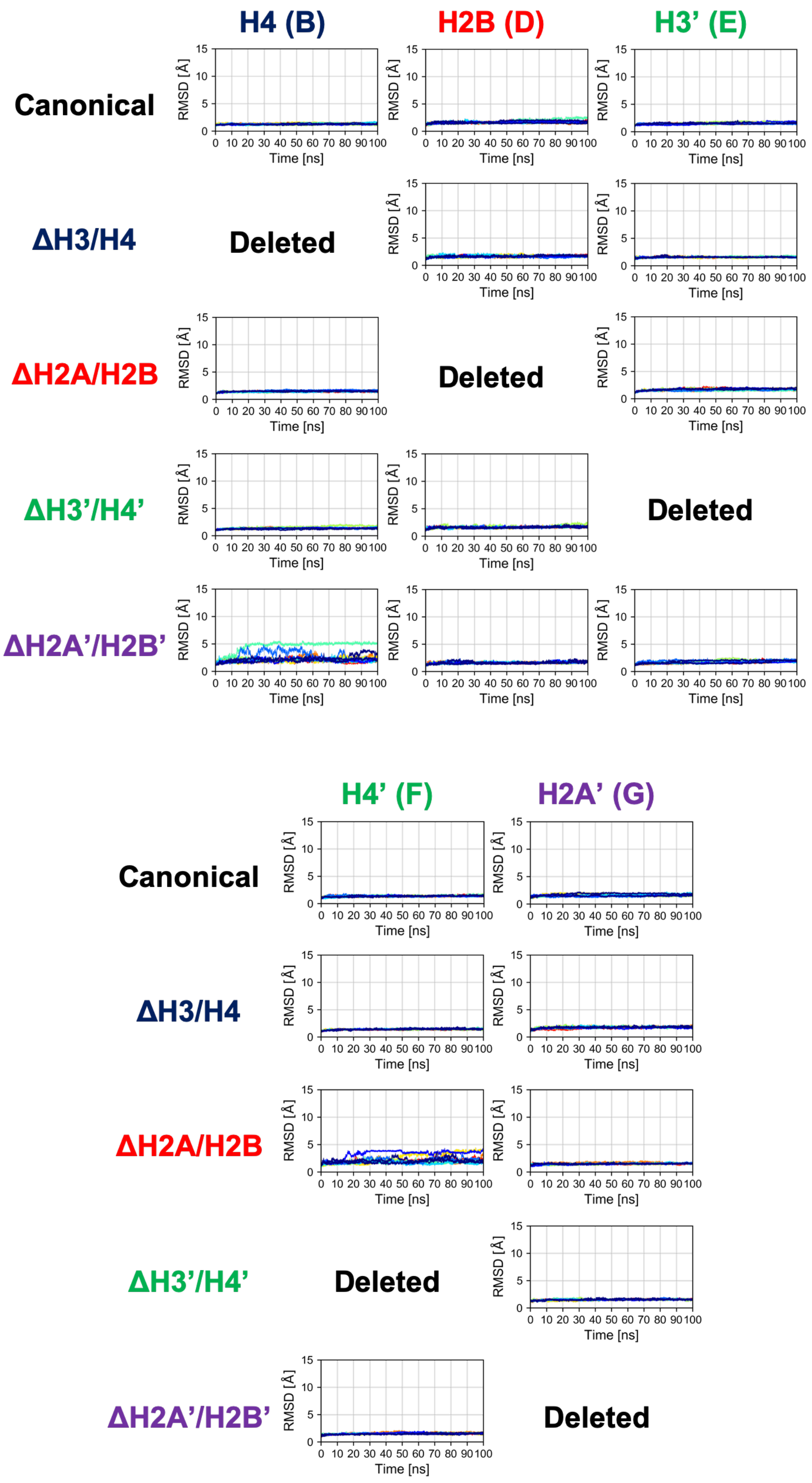

Figure S2. Time Series of RMSD of Histone in Each Nucleosome. Colors show different simulation trajectories (total 10). Note that histone H4 has a short C-terminal region which shows disordered conformation. This region usually interacts with H2A/H2B. When an H2A/H2B is absent, this region loses the interaction with the H2A/H2B and can fluctuate more, which causes the increase of RMSD in H4. The behavior is otherwise unaltered.

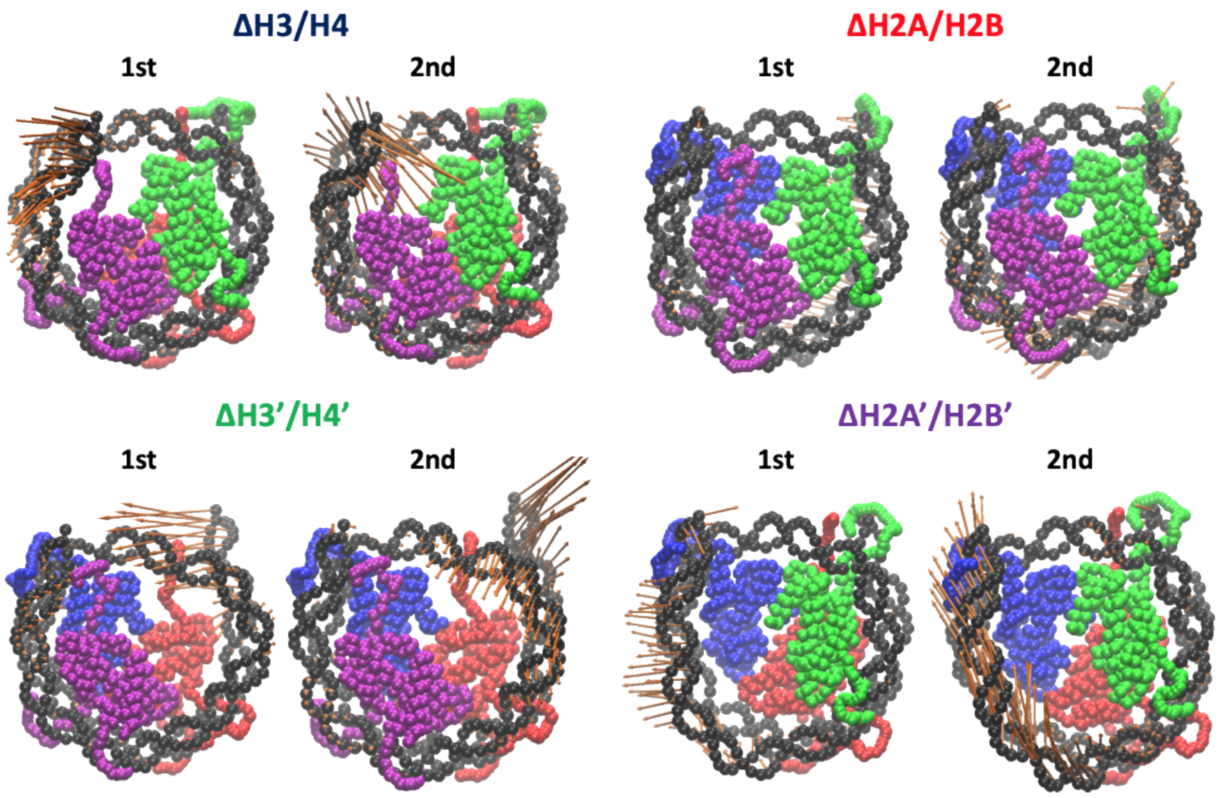

Figure S3. Effective Directions of DNA Deformation (Back View). Visualized from the side of chains E to H (H3', H4', H2A', and H2B'). Nucleotide (C1' of each nucleotide) and amino acid (C^{α} of each amino acid) coordinates are shown by spheres. Orange arrows show the vectors of the 1st and 2nd PC modes. Arrow lengths indicate the norm of the vector.

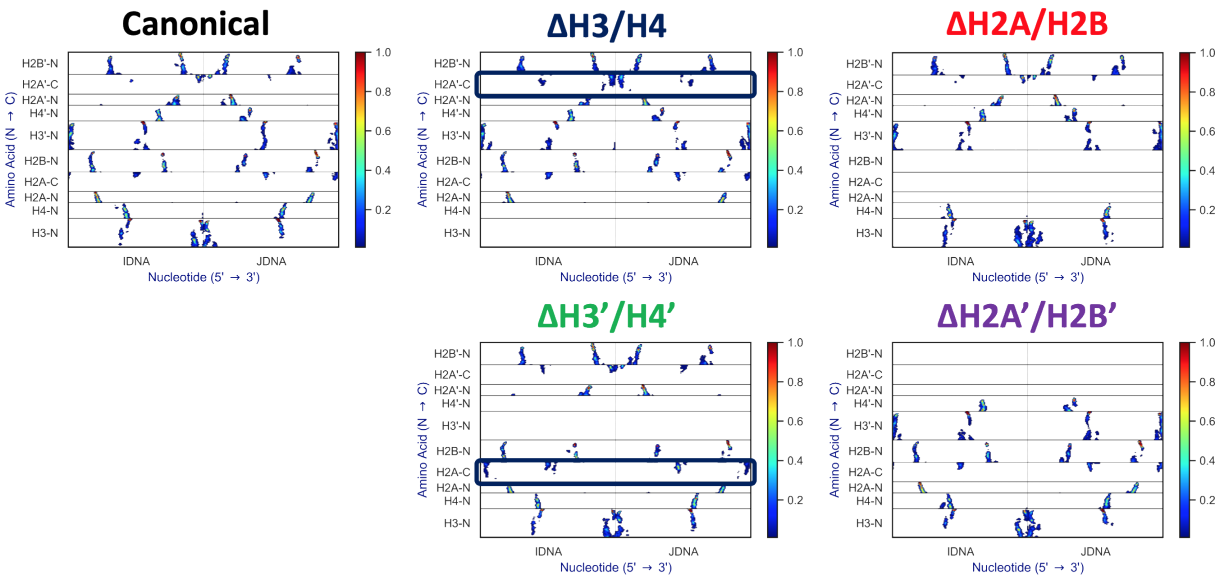

Figure S4. Contact Matrix of DNA and Histone Tails. Contact frequency of nucleotide of DNA and amino acid of histone tails.

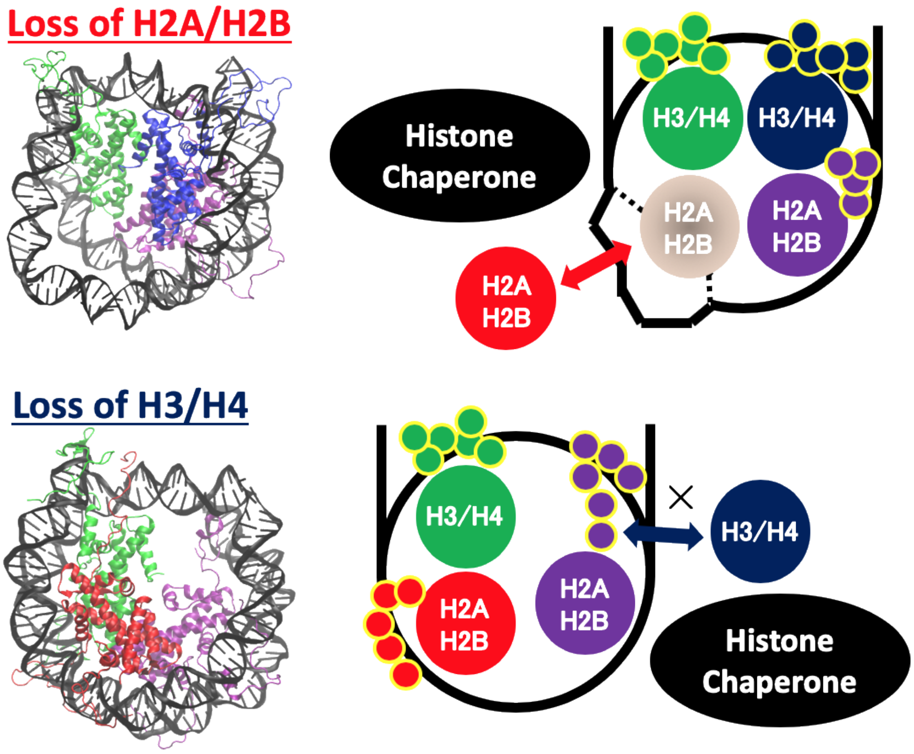

Figure S5. Suggested Schematics of Nucleosome Remodeling based on the Results. Schematics of histone dissociation and nucleosome remodeling by histone chaperone (like FACT). The relationship between partially disassembled nucleosome dynamics and the process of nucleosome remodeling is suggested.
